# Glycemic variability as a risk factor of intensive care unit-acquired weakness

**DOI:** 10.1186/2197-425X-3-S1-A186

**Published:** 2015-10-01

**Authors:** R Mörgeli, T Wollersheim, S Koch, K Haas, M Krebs, S Weber-Carstens

**Affiliations:** Anaesthesiology and Intensive Care, Charité - Universitätsmedizin Berlin, Berlin, Germany

## Introduction

Intensive care unit-acquired weakness (ICUAW) is a serious complication associated with loss of muscle mass and strength, metabolic disorders, organ failure, failure to wean, and increased mortality. ICUAW is diagnosed via MRC scores, and electrophysiological examinations (EP) can detect muscle excitability disorders associated with ICUAW (1). While uncontrolled hyperglycemia and/or insulin resistance has been linked to ICUAW (2), glucose variability, promoted by Krinsley et al as a crucial parameter independently linked to ICU mortality (3), has thus far been neglected in ICUAW studies. There is a lack of standardization in the assessment of glucose variability, and not all methods associate with mortality.

## Objective

The aim was to assess the relationship between blood glucose variability and the incidence of ICUAW. Such a relationship could identify variability as an early ICUAW risk factor. As this was the first attempt to find such correlation, differing metrics were examined.

## Methods

This study examined data from 3 previous studies, including 101 ICU patients from Charité Medical University in Berlin, with a Sequential Organ Failure (SOFA) score ≥ 9 within the first 5 days of admission, mechanical ventilation, available consent, and specific ICUAW diagnostics via MRC and/or EP. Exclusion criteria included diabetes mellitus, BMI > 35, and preexisting neuromuscular disorders. We analyzed 4 daily glucose readings via blood gas analyzer at regular intervals, and computed common variability measurements suitable to our data. (Ethics Vote EA1/017/11)

## Results

**Figure1:**
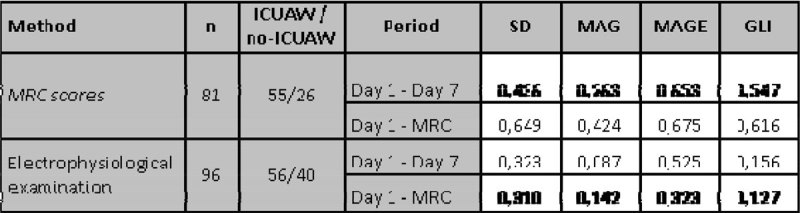
**p-values comparing glucose variability of ICUAW and non-ICUAW patients, grouped by diagnostic method and evaluation perod. Electrophysiological results indicate nonexcitable muscle membrane, but not necessarily ICUAW.**

**Figure 2:**
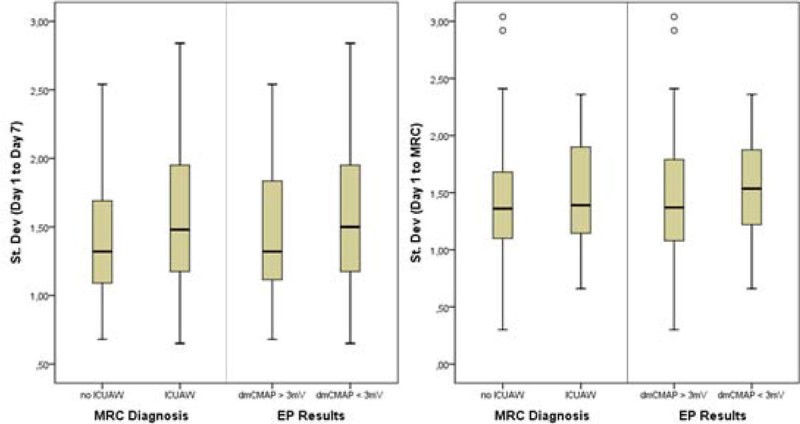
**Patient results for standard deviation, grouped by MRC score (mean <4 = ICUAW) and electrophysiological results (Compound Muscle Action Potential after direct muscle stimulation (dmCMAP < 3mV)) as predictor for ICUAW.**

For each patient, we calculated the standard deviation (SD), mean absolute glucose (MAG), mean amplitude of glycemic excursions (MAGE), and lability index (LI). These values were analyzed against MRC scores and EP diagnoses. Measurements were analyzed for the first 7 days, as well as until the time of the first MRC score, when the patient was deemed sufficiently awake for the first time. No significant correlation and/ or group differences could be found (Figure 1 and 2).

## Conclusions

None of the metrics tested identified a correlation between glycemic variability and ICUAW. Further studies using continuous measurement systems would allow additional metrics to be tested.

## Grant Acknowledgement

Deutsche Forschungsgemeinschaft (DFG), Grant KFO192 WE4386/1-2.
